# Supplementary material for: Chromothripsis during telomere crisis is independent of NHEJ, and consistent with a replicative origin
Source: Genome Res. 2019 May;29(5):737–49. doi: 10.1101/gr.240705.118 (PMC6499312; doi:10.1101/gr.240705.118)
Supplement: Supplemental Material [file supp_gr.240705.118_Supplemental_file_1.zip › contigs/annotated_contigs/DB54/contig.2.DB54_length_538_mean_cov_11.5092936803.docx]

**DB54_length_538_mean_cov_11.5092936803**

```
ATTCTCCTGCCTCAGCCTCCCAAGTAGCTGGGACTACAGGCACCCACCACCACGCCCAGCTAAATTTTTTGTATTTTTAGTATAGACGG
 >chr20:34305884-34306072 + E=2e-101 p=0e+00
GGTTTCACCGAGTTTGCCAGGATGGTCTTGATCTCCTGACCTCGTGATCCACCCGCCTCGGCCTCCCAAA|GTGCTGGGATTACAGGCA
                                                                       >chr19:44021238-4
TGAGCCACTGC|ACCAAGACAAAAGTGATACACTTTCAATAGAAATCATATTCCATGCACCCATACAGCCATTCTGTTTTTCACTTTCA
4021565 + E=3e-179
ATTCAGTATTCAATAAATTACATGAGATATTCAACACTCTTGTATAAAATACAAGGCTTTGTGTTAGATGAGTTTGCCCAACTGTAGGC

TAATGTAAATGTTCTGACCACTTTAAGGTAAAGTAGGCTAACCTATAATGTTCAGTAGGTTAAATGTATTAAATGCGGCCAGGCACGGT

GGCTCACGCCTGTAATCCCAGCACTTTGGGAGGCTGAGGCAGG|CGGATCACAAGGTCAGGAGTTCGAGACCAGTCTGGCCAACATAGT

GAAACAC
```
